# Supplementary material for: Turbulent convection as a significant hidden provider of magnetic helicity in solar eruptions
Source: Sci Rep. 2023 Jun 2;13:8994. doi: 10.1038/s41598-023-36188-z (PMC10238451; doi:10.1038/s41598-023-36188-z)
Supplement: Supplementary file 1 — Supplementary Information. [file 41598_2023_36188_MOESM1_ESM.pdf]

Supplementary Information
A movie for Figure 1 is found at the following URL.
https://www.dropbox.com/s/9b0nn70apt1udda/tile.mov?dl=0
